# Chlorophylls as Natural Bioactive Compounds Existing in Food By-Products: A Critical Review

**DOI:** 10.3390/plants12071533

**Published:** 2023-04-02

**Authors:** Peyman Ebrahimi, Zahra Shokramraji, Setareh Tavakkoli, Dasha Mihaylova, Anna Lante

**Affiliations:** 1 Department of Agronomy, Food, Natural Resources, Animals, and Environment—DAFNAE, University of Padova, Viale dell’Università, 16, 35020 Legnaro, Italy; peyman.ebrahimi@phd.unipd.it; 2 Department of Land, Environment, Agriculture, and Forestry—TESAF, University of Padova, Viale dell’Università, 16, 35020 Legnaro, Italy; zahra.shokramraji@studenti.unipd.it (Z.S.); setareh.tavakkoli@studenti.unipd.it (S.T.); 3 Department of Biotechnology, University of Food Technologies, 26 Maritza Blvd., 4002 Plovdiv, Bulgaria; dashamihaylova@yahoo.com

**Keywords:** antioxidants, natural pigments, food wastes, circular economy approach, sustainability, food dyes, natural colors, functional foods, ultraviolet

## Abstract

Chlorophylls are a group of naturally occurring pigments that are responsible for the green color in plants. This pigment group could have numerous health benefits due to its high antioxidant activity, including anti-inflammatory, anti-cancer, and anti-obesity properties. Many food by-products contain a high level of chlorophyll content. These by-products are discarded and considered environmental pollutants if not used as a source of bioactive compounds. The recovery of chlorophylls from food by-products is an interesting approach for increasing the sustainability of food production. This paper provides insight into the properties of chlorophylls and the effect of different treatments on their stability, and then reviews the latest research on the extraction of chlorophylls from a sustainable perspective.

## 1. Introduction

A considerable quantity of by-products, such as stems, leaves, pulps, and peels, are produced during the cultivation and processing of fruits and vegetables in the food industry and agriculture [1]. These by-products contain high levels of food pigments, specifically chlorophylls, which are present in almost every green part of a crop [2,3,4]. When these by-products are discarded they can become environmental pollutants and lead to the production of a significant quantity of greenhouse gases [5,6]. However, they could serve as a primary sources for the recovery of bioactive compounds, providing health benefits to consumers [7,8,9,10]. Therefore, the recovery of bioactive compounds from these vegetal by-products and reusing them in the production of functional foods can be considered a circular economy approach [11,12,13,14].

Chlorophylls are among the most prominent bioactive compounds and are proven to have many positive impacts on health through their anti-inflammatory and anti-cancer properties [15]. Moreover, it is proven that chlorophylls have a high antioxidant activity [16]. They are used as natural food coloring agents and have wound healing and anti-mutagenic properties [17]. However, despite their health benefits, natural pigments have a variety of downsides, including instability and low utilization [18]. The awareness about the health benefits and the application of chlorophylls as natural food colorants has increased the demand for chlorophyll pigment [3]. Thus, it is important to find possible options for increasing the efficiency in recovering chlorophylls from plants to meet the needs for these precious compounds.

There are various methods for the extraction of chlorophyll, although not all these methods are environmentally friendly. Valorizing food by-products is possible through conventional extraction (CE) and green extraction (GE) techniques. CE methods have many drawbacks, including a low extraction yield, high cost, and the involvement of intense chemicals during a long extraction time, which cause environmental issues and decrease the efficacy of these methods [6,19]. Thus, it is important to undertake the optimum recovery process that includes pre-treatments and extraction methods.

To our knowledge, there appears to be a gap in the literature with regard to a comprehensive review on chlorophylls that takes sustainability into consideration. As such, it is imperative to gather the current information on the recovery of chlorophylls from food by-products and elucidate its nutritional and functional properties. The purpose of this study is to provide a comprehensive review of the chemical composition, extraction methods, bioavailability, and diverse applications of chlorophylls, while also exploring the impact of various treatments on their stability.

## 2. Chemical Composition and Different Types of Chlorophylls

Chlorophylls are based on a porphyrinic structure, comprising four pyrrole rings (C_4_H_4_NH) that are coordinated by a magnesium ion in the central position with a long hydrophobic alkyl chain attached to it [16]. Chlorophylls are examples of porphyrin ring structures with one reduced double bond, called chlorine. At the center of the chlorine is magnesium, which is bonded to the tetrapyrrole ring. The chlorophyll molecules constitute a hydrophilic (porphyrin) group head and a lipophilic hydrocarbon tail (phytol group). Due to their lipophilic hydrocarbon chains as a phytol tail, they are generally considered insoluble in polar solvents [3].

**Figure 1:**
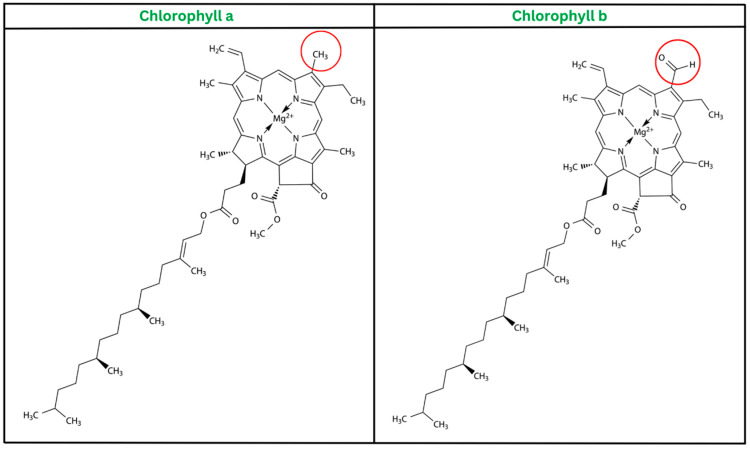
The chemical structure of chlorophyll a and b. The difference is highlighted with a red circle.

Chlorophylls are oil-soluble, amphiphilic pigments with a green color, which are extensively distributed in plants, algae, and cyanobacteria. Chlorophylls can be found in plants with two different structures. Chlorophyll a (C_55_H_72_MgO_5_N_4_) has a methyl (–CH_3_) group at the carbon-7 position, while chlorophyll-b (C_55_H_70_MgN_4_O_6_) has an aldehyde (–CHO) group at the same position [20]. The difference in the chemical structure of chlorophyll a and b is shown in Figure 1. The distinct structural characteristics of the chlorophylls result in variations in their color, with chlorophyll a showing a blue-green color and chlorophyll b showing a blue-yellow color. Chlorophyll a and chlorophyll b coexist in plants at a ratio of 3:1 [20]. According to some other studies, plants that grow in the shade have a higher proportion of chlorophyll b compared to chlorophyll a. This difference was observed in sunlight in some other types of leaves, as well as when a single species was grown under different light intensities [21].

## 3. The Role and Location of Chlorophylls in Plants

**Figure 2:**
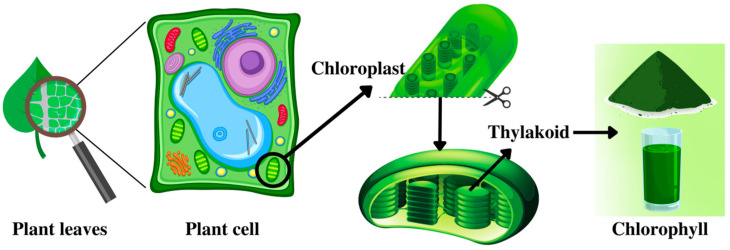
Schematic of the location of chlorophylls in plant cells.

Color pigments are secondary plant metabolites that serve vital roles in plant photosynthesis, such as collecting sunlight, maintaining metabolism, and preventing photo-oxidative disfigurement, among other functions [18]. The term chlorophyll is derived from the Greek words, *chloros* meaning “green” and *phyllon* meaning “leaf” [2]. Chlorophylls are found within the chloroplast, the main organelle which contains the highest amount, in almost every green part of a plant, i.e., the leaves and stem. Chloroplasts are found in the mesophyll layer, in the middle of the plant leaves. Chloroplasts possess thylakoid membranes that contain a green chlorophyll pigment [2]. Figure 2 shows the schematic of chlorophyll inside the plant cells. Environmental conditions such as heat and drought could damage the structure of the chloroplast and decrease the chlorophyll content of a plant [22].

A chloroplast can be referred to as the “food factory” of the plant cell because it produces energy and glucose for the whole plant in association with CO_2_, water, and sunlight. Although there are different photosynthetic pigments, such as carotenoids and phycobilins that entrap solar radiation, chlorophylls are the most important. Chlorophylls convert solar energy into chemical energy that is used to build essential carbohydrate molecules (glucose), which are used as a food source for the whole plant [2]. As shown in Equation (1), during photosynthesis, chlorophylls convert carbon dioxide (CO_2_) and water (H_2_O) into carbohydrates in the presence of light energy.

(1) $${CO}_{2}+H_{2}O\to \left({CH}_{2}O\right)+O_{2}$$

where CH_2_O represents the sugars, carbohydrates and all the cellulose synthesized in the plant [23].

## 4. Extraction of Chlorophyll

The valorization of vegetal by-products could meet the need for the production of chlorophylls on an industrial scale, with applications for producing functional foods, pharmaceuticals, and cosmetics [3]. The valorization processes could be conducted through the extraction of color pigments and other bioactive compounds using the CE (i.e., Soxhlet, maceration, etc.) and GE techniques (i.e., ultrasound, pulsed electric field, etc.) [18]. Although the CE methods can obtain reliable extraction yields, they have several drawbacks, such as a low yield, high cost, and the use of harsh chemicals that can cause environmental harm and decrease the effectiveness of the method [6,19]. Thus, due to the limitations of the CE methods, there is a need for more environmentally friendly techniques to ensure a safe extraction [6]. 

Green techniques for the extraction of color pigments and bioactive compounds from vegetal wastes and by-products include ultrasound-assisted extraction (UAE) and microwave-assisted extraction (MAE). These techniques can positively impact the extraction process, resulting in shorter extraction times and a higher efficiency of both the energy and solvents [24]. Nonetheless, there are still certain disadvantages associated with these techniques, such as their technical intricacy, insufficient management of the energy input, decrease in the levels of bioactive compounds that are sensitive to heat, high initial expenses, and inadequate yield during the extraction process [6].

Table 1 summarizes the different literature on the extraction of chlorophylls from food by-products and other materials. It can be concluded that the efficiency of the extraction could be impacted by various factors, such as the solvent/liquid ratio, temperature, solvent type, concentration, and extraction time. Changing each of these factors can decrease/increase the yield of the chlorophyll extraction depending on the extraction method and the type of material used for the extraction. Thus, it is important to find the optimized extraction condition to achieve the highest yield for the recovery of chlorophylls from food by-products.

Elevating the temperature (no higher than 50 °C) can enhance the process of extracting chlorophylls from the material. Since chlorophylls are sensitive to high temperatures, when the extraction temperature exceeds 60 °C, the chlorophylls undergo a conversion into a different compound called pheophytin, which results in a decrease in the amount of total chlorophyll in the sample. The conversion of chlorophyll to pheophytin occurs through the replacement of magnesium, which is mainly performed by acidic substitution, heat treatment, or after the action of Mg chelatase [2].

The solvent type can affect the chlorophyll extraction. Chlorophylls are usually extracted using organic solvents such as diethyl ether, ethanol, dimethyl sulfoxide, acetone, and methanol [27]. The typical solvent extraction relies on the penetration capability of the solvent to the cell membrane for dissolving the lipids as well as the lipoprotein of the chloroplast membranes [17]. Green extraction techniques involve solvents such as ionic liquids, ethanol, esters of fatty acid or oils of fruits and vegetables (soybean, rapeseed oil, cocoa oils etc.), glycerol etc., which have all gained importance in the extraction methods of natural pigments [24]. Ferreira et al. (2021) extracted chlorophylls from spinach leaves using an aqueous solutions of surface-active ionic liquids. The extraction process was successful and yielded good results, especially when using hexadecylpyridinium chloride. However, it is noteworthy to mention that these types of solvents are toxic and the extracts obtained using them could not be used for human consumption [31]. Lee et al., 2021 used 2,3-butanediol as a solvent for the extraction of chlorophyll a from *Nannochloropsis* sp. They reported that the extraction yield with this solvent could reach 91.8%. However, the extraction using 2,3-butanediol is not considered green processing as this solvent could be toxic. Another limitation could be the high energy-consuming production process of 2,3-butanediol [32].

It was reported that acetone is the best solvent, in terms of efficiency, for the extraction of chlorophylls [27]. Acetone is used widely as the extracting solvent in the extraction of chlorophylls. However, it is not a safe solvent due to its high flammability, and its adverse effects on the human body, including headache, and erythema (i.e., skin irritation), even when used with plastic or latex gloves. Methanol is a very good extractant for chlorophylls. It is less volatile and flammable than acetone but is notoriously toxic. Ethanol is a much safer solvent than either acetone or methanol [33]. The extraction with methanol results in a very unstable solution, which stimulates the formation of the product degradation. However, ethanol could yield good results, and it has a green nature, which makes it a preferable solvent for the extraction of chlorophylls [17]. Although water could not be used for the extraction of chlorophylls since they are oil-soluble compounds, it could be combined with organic solvents during the extraction process.

There are also some pretreatments, such as microwave, ultrasound, homogenization, grinding, etc., that can enhance the yield of the extraction process [17]. It is reported that drying can increase the chlorophyll content of the leaves [34]. Ferreira et al. (2020) applied microwave blanching before the freeze-drying process in broccoli by-products and found that blanching increased the extractability of chlorophylls by inactivating the chlorophyll-degrading enzymes [35]. Derrien et al. (2018), utilized a green extraction technique assisted with a supercritical CO_2_ extraction using 93% ethanol for the extraction of chlorophylls and lutein from spinach by-products/wastes. They confirmed a higher recovery of phytopigments (70% for lutein and 96% for chlorophylls) when compared to the conventional extraction using acetone [15]. Moreover, Sarkar et al. (2020) extracted chlorophylls from isolated *Chlorella thermophila* using ethanol as a solvent, and they reported that boiling the cells (at the boiling point of the solvent under reflux) for 3−5 min before the extraction resulted in the complete extraction of the chlorophylls without any degradation. However, boiling at a temperature of 100 °C can induce the degradation of chlorophylls [17].

## 5. Effect of Different Treatments on Chlorophylls

The degradation of chlorophylls poses a significant challenge for preserving the quality of vegetables and fruits during post-harvest storage. Chlorophylls are mainly degraded by several enzymes, including chlorophyllase, Mg-dechelatase, pheophytinase, peroxidase, and chlorophyll oxidase, which are responsible for breaking down the chlorophyll molecules [36]. The removal of phytol and Mg using chlorophyllase and Mg-dechelatase are the two main steps in the degradation of chlorophylls [2]. 

The degradation of chlorophylls can result in a loss of color, texture, and nutritional value in food products, making it crucial to develop effective post-harvest management strategies [36]. On the other hand, the color of chlorophylls in the extracts recovered from green plants is a negative attribute since it interferes with the measurement of the other compounds and makes it difficult to use the extract inside the foods. In order to solve these issues, various decolorization techniques can be employed, such as utilizing activated charcoal to absorb the chlorophylls present in the sample, or breaking down the chlorophyll through exposure to UV radiation [23,37]. There are different treatments that can impact the content of chlorophylls by promoting oxidation and affecting the chlorophyll-degrading enzymes and antioxidative compounds. Table 2 shows the effects of the different treatments on the chlorophylls present in the plants and their by-products.

Blanching has the potential to trigger the production of chlorophyll-derived substances, primarily caused by the removal of Mg^2+^ from the chlorophyll to create pheophytin. Blanching also leads to the formation of other chlorophyll-derived substances, such as pyropheophytin a, pheophorbide a, chlorophyllides a and b, and pyrophaeophorbide a, which are typically associated with the thermal processing. However, blanching could increase the inactivation of chlorophyll-degrading enzymes, which can positively impact the extraction process [35].

UV radiation causes the chlorophyll molecules to become uncoupled in the light-harvesting system, leading to a decrease in photosynthesis in various plant species. In many cases, even small doses of UV radiation cause a shift in the ontogenetic sequence of the photosynthetic capacity. UV radiation causes dramatic changes in the biomass production, leaf development, stomas. All these changes directly affect photosynthesis [45,46]. During the early stages of exposure to UV-C radiation, the rate of degradation of the chlorophyll exceeds the rate of synthesis. However, as the exposure continues, the synthesis of the chlorophylls may increase to provide better protection to the plant [47,48]. The literature shows that melatonin can regulate the production of the photosynthetic and defensive pigments, such as chlorophylls helping to maintain the photosynthetic apparatus under light stress and conditions such as temperature stress, which reduce the efficiency of the photosynthetic apparatus [49].

Chairat et al. (2013) reported that a UV-C radiation treatment of Chinese kale resulted in an enhanced stability of the chlorophylls, postponed leaf yellowing, and reduced the activity of the enzymes [50]. Studies have shown that UV-B and UV-C treatments can delay the chlorophyll degradation during storage. However, the effectiveness of the UV-C treatment may vary depending on the environmental factors, such as the temperature [51,52]. Another investigation was conducted on broccoli (*Brassica oleracea* L. *var. italica*). Green LED lighting enhanced the chlorophyll content of the broccoli, while red and yellow LED lighting increased the phenolic compound content and improved the overall appearance of the broccoli florets compared to the control [53]. A study found that blue and UV-A lights of different wavebands worked synergistically to increase the accumulation of the chlorophylls in Chinese kale and pakchoi baby leaves [54]. It was reported that UV-A and UV-B lights were effective for increasing the chlorophyll content, and blue light enhanced the chlorophyll synthesis in various leafy greens [55]. Kaewsuksaeng et al. (2011) reported that UV-B irradiation neutralized the activity of chlorophyll-degrading enzymes, chlorophyllase, Chlorophyll-degrading peroxidase, and pheophytinase in stored lime (*Citrus latifolia Tan*.) fruits [36]. UV-C was especially effective for inhibiting the chlorophyll degradation in stored broccoli florets [56]. According to Aiamla-or et al. (2010), the degradation of the chlorophylls in broccoli during storage was effectively delayed using UV-B treatment [52]. Similarly, Srilaong et al. (2011) found that UV-B treatment was successful in slowing the decrease in the levels of the chlorophyll derivatives in mature green limes during storage [57]. The reduction in the chlorophyll degradation using UV stemmed from its ability to remove the chlorophyll-degrading enzymes. However, the discoloration of the chlorophylls under the exposure to light was due to the degradation of chlorophyll a into small molecular compounds. In addition, singlet oxygen (^1^O_2_) was generated through photosensitive reactions when chlorophyll a was irradiated with light, and this also caused a chlorophyll a discoloration [58].

Moreover, electron beam irradiation is another technology that can help prolong the shelf life of vegetables. Pongsri et al. (2021) reported that treating lime peel with an electron beam maintained the total chlorophyll content and suppressed the activity of the chlorophyll-degrading enzymes [59]. The degradation of chlorophylls in electron-beam-treated mango was found to be lower than the control at the end of the storage period [38]. 

## 6. Nutritional Properties and Health Benefits of Chlorophylls

Several beneficial properties have been linked to green vegetables, such as antioxidants, anti-mutagens, and detoxifications [60]. Regularly consuming green-colored vegetables, such as spinach or cruciferous vegetables, has been shown to relieve the risk of chronic disease [61]. Phytonutrients, specifically plant pigments such as chlorophylls, carotenoids, and betalains, were previously regarded for their technological use as colorants. However, they are now the subject of research for their exceptional nutritional properties. Chlorophylls and their derivatives have shown important health-promoting functions, showing anti-mutagenic, anti-cancer, and anti-inflammatory activities [5]. Studies suggest that chlorophylls are rich sources of vitamins E, A, C, K, and β-carotene, along with essential minerals such as magnesium, potassium, iron, calcium, and essential fatty acids [52]. 

It was reported that incorporating chlorophyll into the diet during early life stages can help decrease weight gain, improve glucose tolerance, and lower inflammation, which can prevent obesity [62]. Moreover, the effect of chlorophyll on rats with type one diabetes was investigated, and it was confirmed that chlorophyll a can decrease the risk of diabetes [63]. Chlorophyll extracts from mint, broccoli, thyme, and bell peppers may aid in blood sugar control [64]. It was also reported that phytol, which is a product from the degradation of chlorophylls, can reduce joint inflammation and pain by inhibiting the inflammatory mediators [65].

Moreover, chlorophyll pigment in green varieties with dark colors can have a protective effect against specific cancers, such as colon and liver cancers. The mechanism of chlorophyll binds together hydrocarbons, aflatoxins, and other hydrophobic molecules that are related to cancer, rather than eliminating them [66].

## 7. Application of Recovered Chlorophylls in Functional Foods

Plant pigments are unique chemical compounds that give fruits and vegetables a vibrant color and an attractive look. Natural coloring pigments, which are an excellent source of color, could replace many synthetic food colorings. Various types of natural pigments are available in nature such as chlorophylls, anthocyanins, carotenoids, betanin, flavonoids, quinones, and xanthones [18]. The extracts of chlorophylls (or chlorophyll derivatives) and the copper complexes of chlorophylls are known as natural colorants with E-numbers of E140 and E141, respectively. Chlorophylls are marketed as “E140i” directly after the solvent extraction from edible plants. Whereas chlorophyllin, marketed as “E140ii”, is obtained through the saponification of chlorophylls and has a stable color [17]. The application of chlorophyll, if obtained from edible plants, grass, and nettles, is allowed in Europe [67].

**Figure 3:**
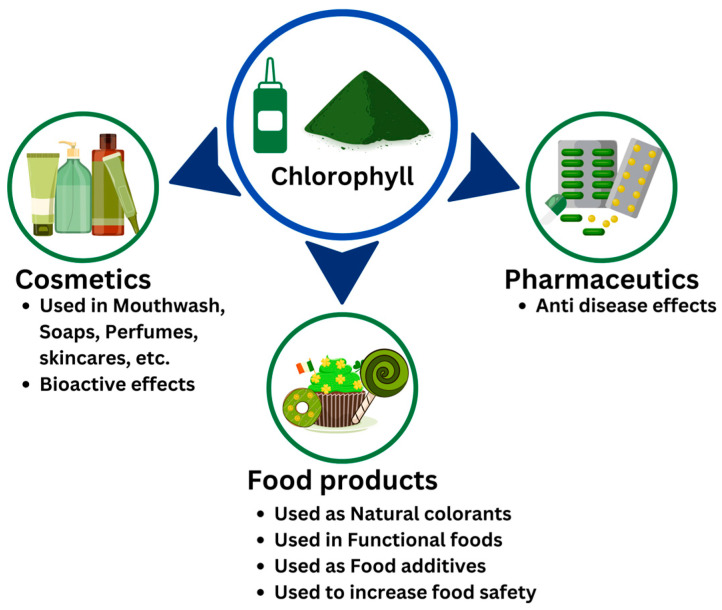
Different applications of chlorophyll dye.

Green food by-products are rich sources of chlorophylls that offer both coloring and health-promoting properties both in foods and cosmetics as well as their application in pharmaceuticals owing to its bioactive properties [16,20,27,68]. Therefore, the chlorophyll recovered from these by-products could be reused in several applications, as shown in Figure 3. 

The concern about healthcare among consumers has increased the market demand for healthy and safe foods [69]. Many efforts have been made to meet this need through the development of functional foods. Adding bioactive compounds to food products to produce functional foods can enhance their health benefits [70]. Zen et al. (2020) added microencapsulated *spirulina,* which is a rich source of chlorophylls to pasta in order to increase its antioxidant properties [71,72]. Batista et al. (2017) utilized microalgae biomass, which has a high content of chlorophylls, in cookies to increase their bioactive active compounds [73]. 

It was also reported that chlorophylls could have a good anti-microbial effect [26]. Elbatanony et al. (2019) investigated the anti-microbial effect of the pigment extracts of *Punica granatum* L. leaves, which had a total chlorophyll of 4.9 ± 0.251 mg/g. Their results highlighted that 150 μL of the pigment extracts at 60 min could inhibit different types of bacteria, yeast, and fungus [74]. Pothiraj et al. (2021) reported that the extracts obtained from *Acanthus ilicifolius* L. and *Heliotropium curassavicum* L. plants at a concentration of 50 μg/mL could inhibit the growth of bacterial pathogens. These extracts had a considerable quantity of chlorophylls [75]. Moreover, Dziedziński et al. (2020) co-extracted chlorophylls and phenolic compounds from *Pinus sylvestris* L. shoots and evaluated the anti-microbial activity of the obtained extracts. They reported that the extracts could efficiently prevent the growth of gram-negative bacteria [76]. However, it should be noted that the presence of phenolic compounds inside of the pigment extracts could be a synergist in the anti-microbial activity, as polyphenols are known to have anti-microbial properties [77]. 

Natural chlorophylls consisting of both chlorophyll a and chlorophyll b are approved as food additives [17]. Modifying and enhancing traditional foods with natural health-promoting ingredients can help consumers counteract diet-related illnesses and adopt a healthier diet. These modified foods, known as functional foods, are particularly beneficial because they contain increased concentrations of bioactive compounds, such as secondary plant metabolites. These compounds offer important health benefits and are believed to play a key role in promoting overall well-being [78]. 

Using natural colorants as food additives can enhance the sensory attributes and nutritional properties of food products [79]. Jayasinghe et al. (2016) used the chlorophyll extract obtained from seaweed in the preparation of jelly dessert. They reported that the color of the prepared jelly dessert stayed constant for more than 30 days at an ambient temperature [80]. However, using chlorophylls as coloring additives could be difficult due to their instability when exposed to different food components, temperatures, light, oxygen, pH, packaging materials, and storage conditions. [79]. A possible method to increase the stability of chlorophylls is using an appropriate nano-formulation technique such as encapsulation. Liu et al. (2021) prepared a chlorophyll nano-emulsion from pomelo leaves and reported that the proposed method could yield in a highly stable chlorophyll, which could be used for its health beneficial effects [81].

By replacing the central magnesium in the structure of chlorophylls with copper, a semi-synthetic colorant called chlorophyllin can be synthesized, which has a stable color and is water-soluble. This pigment could be commercially used in the food industry as a stable green coloring agent [67]. Paskeviciute et al. (2019) used chlorophyllin to reduce the microbial load in basil. They reported that soaking the basil in chlorophyllin and exposing it to light at 405 nm could be a method to increase the safety and shelf life basil [82].

## 8. Conclusions

The recovery of chlorophylls from food by-products is crucial for harnessing the bioactivity of this compound in food products and adopting a circular economic approach. A growing body of research explored the extraction of chlorophylls from food by-products, but a major challenge remains in the insolubility of the chlorophylls, making it difficult to use water as an extracting solvent. As such, future research efforts in this area should focus on developing treatments that can enhance the solubility of chlorophylls, allowing for the use of water as a green solvent for its extraction.

Moreover, using natural colorants as coloring additives can be challenging due to their instability when exposed to various factors such as temperature, light, and storage conditions. Therefore, further research is needed to enhance the stability of natural colorants. These efforts would contribute to the development of sustainable and environmentally friendly methods for extracting bioactive compounds from food waste.

Overall, the findings of this review highlight the importance of exploring novel and sustainable approaches for recovering valuable compounds from food by-products, which can contribute to the promotion of a circular economy and reduce environmental waste.

## Figures and Tables

**Table 1:** Extraction of chlorophylls from different plant tissues.

Material	Extraction Method	Solvent	Temp (°C)	Extraction Time (min)	Solid-to-Liquid Ratio (g:mL)	TCC ^1^ (mg/g)	Ref.
*Chlorella vulgaris* residue	UAE ^2^	EtOH 79.4%	61.4	78.7	50:10	31.1 ± 1.56	[25]
Alfalfa *(Medicago sativa* L.) leaves	UAE	EtOH 96%	35	60	1:10	1.74	[26]
Biomass of *Chlorella thermophila* isolate	High-speed homogenizer	EtOH 96%	58	6	1:1	60.41	[17]
Spinach leaves	CE ^3^	Aqueous Solutions of Nonionic Surfactants	41	30	7:1000	0.94 ± 0.03	[16]
Biomass of *Arthrospira platensis*	CE	EtOH 100%	27	720	1:5	5.75	[27]
Pandan leaf	MAE ^4^	Acetone 100%	-	2	1:30	0.42	[21]
Biomass of *Chlorella vulgaris*	CE	EtOH 95%	22–25	30	1:5	15.4	[28]
Spinach by-products	CE	Acetone 100%	25	20	05:10	1.13	[29]
Kiwi Juice Pomace	MAE	EtOH 50%	75	15	1:15	0.06	[30]

The units may be converted to make the comparison possible. ^1^ Total chlorophyll content; ^2^ ultrasound-assisted extraction; ^3^ conventional extraction; ^4^ microwave-assisted extraction.

**Table 2:** The effect of different treatments on the color and chlorophylls.

Materials	Treatment	Condition	Effects	Result	Ref.
Mango	Electron beam	Treatment with 0.5 kGy electron beam	Decreased pheophytinase and peroxidase activity	Decrease in the degradation of chlorophylls	[38]
Grape leaves	UV-C	245 nm, 15 W, 10 min, distance: 12.5 cm	Increased the reactive oxygen species	Decrease in the chlorophyll content	[39]
Fresh-cut stem lettuce	UV-C	254 nm, intensity: 16.6 W m^−2^, irradiation: 8 kJm^−2^, distance: 20 cm	Reduced the activity of chlorophyllase and Mg-dechelatase	Decrease in the chlorophyll degradation	[40]
Broccoli florets	UV-B	310 nm, intensity: 20.4 Wm^−2^, irradiation: 1.2 kJm^−2^	Reduced the activities of chlorophyllase and pheophytinase	Delay in the yellowing of the broccoli florets	[41]
Pineapple	UV-C	Irradiation: 26.4 kJm^−2^	Increased total phenolic content and antioxidant activity (DPPH and FRAP	Increase in the maintenance of the color characteristics	[42]
Broccoli florets	Purple LED	The light intensity was approximately 40 µmols^−1^m^−2^	Downregulated the expression of the genes related to the chlorophyll degradation	Increase in the stability of chlorophyll	[43]
Strawberry	Elevated CO_2_	Treatment with air containing 20% CO_2_	Inhibited chlorophyllase and Mg-dechelatase activity	Delay in the degradation of chlorophylls	[44]

## Data Availability

Not applicable.
